# Calcium sparks in the intact gerbil spiral modiolar artery

**DOI:** 10.1186/1472-6793-11-15

**Published:** 2011-08-26

**Authors:** Gayathri Krishnamoorthy, Keil Regehr, Samantha Berge, Elias Q Scherer, Philine Wangemann

**Affiliations:** 1 Anatomy & Physiology Department, Kansas State University, Manhattan, Kansas 66506, USA; 2 ENT-Department, Technical University of Munich, Munich, Germany; 3 Department of Biomedical Engineering, University of Wisconsin-Madison, Madison, WI, USA

## Abstract

**Background:**

Calcium sparks are ryanodine receptor mediated transient calcium signals that have been shown to hyperpolarize the membrane potential by activating large conductance calcium activated potassium (BK) channels in vascular smooth muscle cells. Along with voltage-dependent calcium channels, they form a signaling unit that has a vasodilatory influence on vascular diameter and regulation of myogenic tone. The existence and role of calcium sparks has hitherto been unexplored in the spiral modiolar artery, the end artery that controls blood flow to the cochlea. The goal of the present study was to determine the presence and properties of calcium sparks in the intact gerbil spiral modiolar artery.

**Results:**

Calcium sparks were recorded from smooth muscle cells of intact arteries loaded with fluo-4 AM. Calcium sparks occurred with a frequency of 2.6 Hz, a rise time of 17 ms and a time to half-decay of 20 ms. Ryanodine reduced spark frequency within 3 min from 2.6 to 0.6 Hz. Caffeine (1 mM) increased spark frequency from 2.3 to 3.3 Hz and prolonged rise and half-decay times from 17 to 19 ms and from 20 to 23 ms, respectively. Elevation of potassium (3.6 to 37.5 mM), presumably via depolarization, increased spark frequency from 2.4 to 3.2 Hz. Neither ryanodine nor depolarization changed rise or decay times.

**Conclusions:**

This is the first characterization of calcium sparks in smooth muscle cells of the spiral modiolar artery. The results suggest that calcium sparks may regulate the diameter of the spiral modiolar artery and cochlear blood flow.

## Background

The gerbil spiral modiolar artery (SMA) originates via the anterior inferior cerebellar artery from the basilar artery and provides the blood supply to the cochlea. It has an outer diameter of ~60 μm and follows the eighth cranial nerve from the brain stem to the modiolus of the cochlea [1]. The SMA is an end-artery that feeds the capillary networks of the spiral ligament and the stria vascularis, which maintains the endocochlear potential essential for hearing [2]. This energy-intensive mechanism renders the cochlea vulnerable to ischemia, which is thought to be involved in the pathogenesis of hearing loss and tinnitus. Consequently, the mechanisms that regulate the diameter of the SMA and thereby cochlear blood flow are of great interest.

Vascular tone is determined by the contractility of the smooth muscle cell, which is regulated by membrane-potential and Ca^2+^-dependent as well as independent mechanisms [3]. An important regulator of smooth muscle contractility is the ryanodine receptor (RyR) mediated "Ca^2+ ^spark". Ca^2+ ^sparks are the physical manifestation of coordinated openings of clustered RyRs causing a highly localized and transient increase in the Ca^2+ ^concentration in the subsarcolemmal space [4]. Ca^2+ ^sparks have been demonstrated in all muscle cells - cardiac, skeletal as well as smooth muscle cells. In cardiac and skeletal muscle cells, tight coupling between sarcolemmal voltage-dependent Ca^2+ ^channels (VDCCs) in the T-tubules and RyRs in the terminal cisternae generates a depolarization-induced Ca^2+^-induced-Ca^2+^-release (CICR) process that causes contraction [4]. On the other hand, in smooth muscle cells, particularly in vascular smooth muscle cells, RyRs, large-conductance calcium- and voltage-activated K^+ ^(BK) channels and VDCCs have been shown to form a functional triad that maintains or mediates vasodilation by limiting Ca^2+ ^influx via VDCCs [5-8]. Increases in the Ca^2+ ^concentration in the subsarcolemmal space caused by Ca^2+ ^sparks, which engulf the cytosolic face of BK channels, cause activation of these channels and hyperpolarization of the membrane potential, closure of VDCCs and vasodilation via a decrease in the cytosolic Ca^2+ ^concentration in the vicinity of the contractile myofilaments. Thus, Ca^2+ ^sparks form a negative feedback mechanism that regulates vascular tone and hence blood flow. This mechanism has not yet been identified in the regulation of cochlear blood flow. Previous studies from our lab have indicated the presence of a ryanodine-sensitive Ca^2+ ^sensing receptor in vascular smooth muscle cells that regulates the contractility of the gerbil SMA [9]. A role for RyR-mediated Ca^2+ ^release in hyperpolarization of smooth muscle cells, mediated by activation of BK channels, has been previously suggested in guinea-pig SMA [10].

The goal of the present study was to detail the presence and properties of Ca^2+ ^sparks in smooth muscle cells of the intact gerbil spiral modiolar artery. To that end, a protocol for the measurement of Ca^2+ ^sparks in the gerbil SMA was established and software for the analysis of Ca^2+ ^sparks was developed. We demonstrate for the first time, a detailed analysis of the kinetics and properties of Ca^2+ ^sparks in smooth muscle cells of the intact gerbil SMA and their regulation by pharmacological activators and inhibitors of RyRs as well as changes in smooth muscle membrane potential. The data suggest that Ca^2+ ^sparks are important for regulating the contractility of the smooth muscles of the SMA and thereby vascular tone and cochlear blood flow.

## Results

### Localization of ryanodine receptors

**Figure 1:**
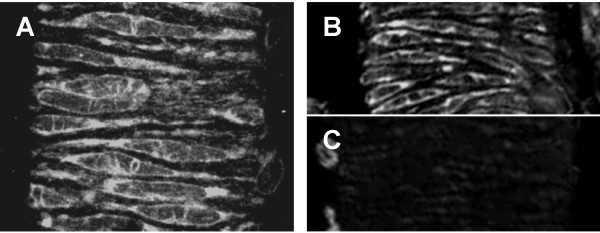
**Staining for ryanodine receptors in the spiral modiolar artery**. **(A) **Ryanodine receptors were labeled with 1 μM Bodipy^®^-ryanodine. Specificity was evaluated by staining with 1 μM Bodipy^®^-ryanodine in the absence **(B) **or presence **(C) **of 250 μM unlabelled ryanodine.

In the first series of experiments, ryanodine receptors were visualized using a green fluorescent derivative of ryanodine. A punctuate staining pattern was expected if ryanodine receptors were limited to or concentrated at distinct spark sites. Staining with 1 μM Bodipy^® ^FL-X ryanodine produced a pattern that was consistent with RyRs being uniformly expressed in the sarcoplasmic reticulum (Figure 1). Specificity of staining was verified by competition with unlabeled ryanodine. Nearly no staining was observed with 1 μM Bodipy^® ^FL-X ryanodine in the presence of 250 μM unlabelled ryanodine. No evidence for a concentration of ryanodine receptors to distinct spark sites was obtained. The staining pattern is consistent with the theory of loose coupling between plasmalemmal L-type Ca^2+ ^channels and sarcoplasmic RyRs in smooth muscle as against the direct spatial and physical coupling observed in cardiac and skeletal muscle respectively [11].

### Detection of Ca^2+ ^sparks

**Figure 2:**
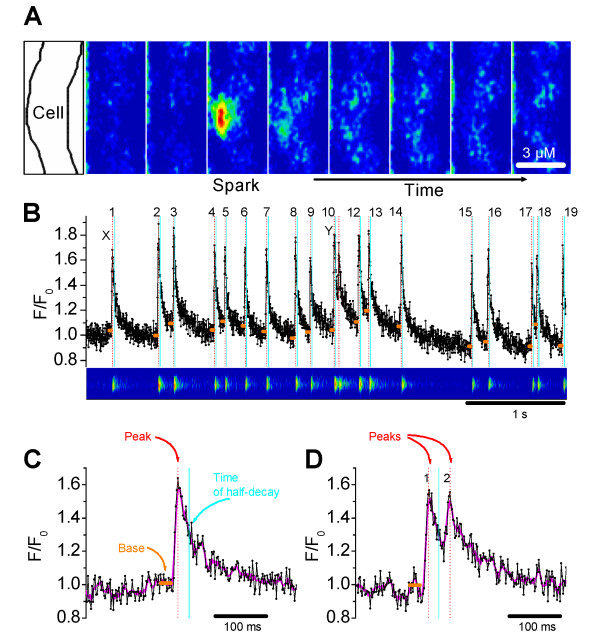
**Imaging and recording of Ca^2+ ^sparks**.** (**A**)** Series of confocal images obtained at a temporal resolution of 41.5 ms. Ca^2+ ^sparks were identified as highly localized elevations in the cytosolic Ca^2+ ^concentration of the smooth muscle cell (outlined in the left-most image).****(B**)** Image and fluorescent trace obtained from a typical 5s scan at a temporal resolution of 1.92 ms shows spontaneous Ca^2+ ^sparks occurring with high frequency. Numbers beside transients indicate sparks. Sparks were identified and frequency was calculated using custom-designed software. The sparks marked 'x' and 'y' are shown in ****(C**)** and **(D) **with an expanded resolution. Amplitude (Peak - Base intensity), rise time (Peak - Base time) and time to half decay (Half decay - Peak time) were obtained from individual Ca^2+ ^sparks. ******(C**) ****An example showing identification of sparks and calculation of spark parameters by the software for a Ca^2+ ^spark rising from the baseline. **(D)** An example showing identification of sparks and calculation of spark parameters by the software for a Ca^2+ ^spark "pair", where the second spark rises before the first returns to baseline. Spark amplitude, rise time and decay time are calculated only from the first and not the second spark.

In a typical recording of Ca^2+ ^sparks from a smooth muscle cell of the intact SMA loaded with the Ca^2+ ^indicator fluo-4, Ca^2+ ^sparks occurred spontaneously (Figure 2). Representative image frames recorded at 41.5 ms intervals show a Ca^2+ ^spark occurring at 124.5 ms after the start of recording (frame 3 of Figure 2A), that rapidly decayed within a single frame interval of 41.5 ms (frame 4). Ca^2+ ^sparks occupied a mean spatial width of 2.4 ± 0.1 μm (n = 25 spark sites). For further analysis of sparks, nine consecutive line-scans, each lasting 5s with a 20s interval between scans, were recorded at selected spark sites. There was no loss of fidelity in the observed Ca^2+ ^sparks under control conditions for the duration of the nine line-scans. Figure 2B shows a typical 5s line scan recording of a chosen spark site where robust Ca^2+ ^sparks occurred with high frequency. Custom written software described in methods proved very effective in detecting and analyzing Ca^2+ ^sparks and spark kinetics from line scans (Figure 2C and 2D). Spark parameters were averaged over the entire recording period. In this preparation under control conditions, Ca^2+ ^sparks occurred with a frequency of 2.6 ± 0.1 Hz/spark site (n = 105 spark sites), with a rise time of 17 ± 0.3 ms and a time to half decay of 21 ± 0.7 ms (averaged from 4,103 sparks recorded from 105 spark sites).

### Effect of ryanodine and caffeine on Ca^2+ ^sparks

**Figure 3:**
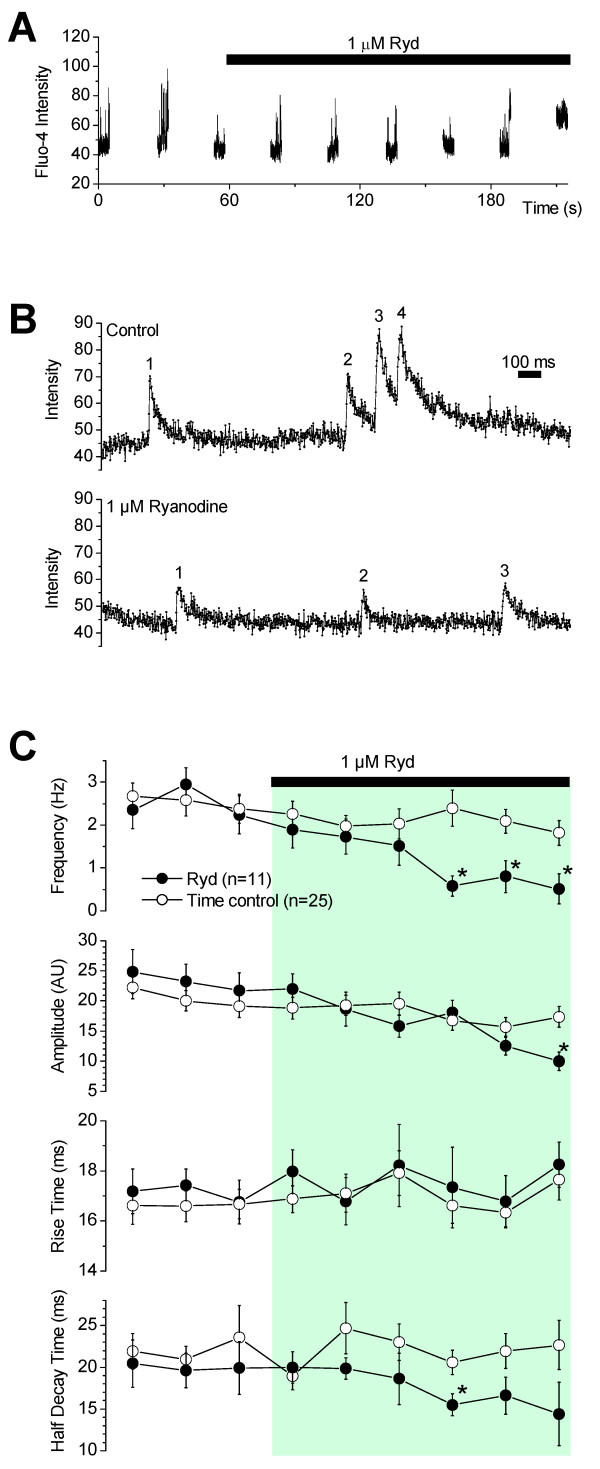
**Ryanodine reduces the frequency of Ca^2+ ^sparks**. **(A) **A typical experiment for the effects of ryanodine on sparks. Ryanodine decreased spark frequency by the 9^th ^scan. **(B) **Expanded line scans from **(A) **shows a trace in control (scan 1) and in ryanodine (scan 9). Numbers beside transients indicate sparks. **(C) **Ryanodine reduced the frequency of Ca^2+ ^sparks but had no major effect on Ca^2+ ^spark rise time or time to half-decay. Recordings in the presence of ryanodine were made at 10, 40, 70, 100, 130 and 160s after application of ryanodine. Time control experiments revealed that Ca^2+ ^spark frequency, amplitude, rise time and time to half-decay were reasonably stable for nine 5 s line scans spread over 225s.

Ryanodine is a plant alkaloid that specifically binds to RyRs and inhibits them at micromolar concentration, leading to the cessation of Ca^2+ ^sparks. A typical line-scan experiment for the application of ryanodine is depicted in Figure 3A. 1 μM ryanodine caused a significant decrease in Ca^2+ ^spark frequency and amplitude by scan 9 (Figure 3B). In five experiments, there was complete cessation of sparks before the end of the experiment. Ca^2+ ^spark frequency in the presence of ryanodine, averaged from 11 experiments, was 0.6 ± 0.3 Hz/spark site, which is a ~4-fold decrease to that observed without ryanodine (Figure 3C). Interestingly, ryanodine did not have a significant effect on the rise-time or decay time of sparks. Rise time and decay time were 17 ± 1 ms and 14 ± 2 ms respectively in the presence of ryanodine (averaged from 104 sparks recorded from 8 spark sites) and 18 ± 0.6 ms and 21 ± 3 ms respectively in time-matched controls.

**Figure 4:**
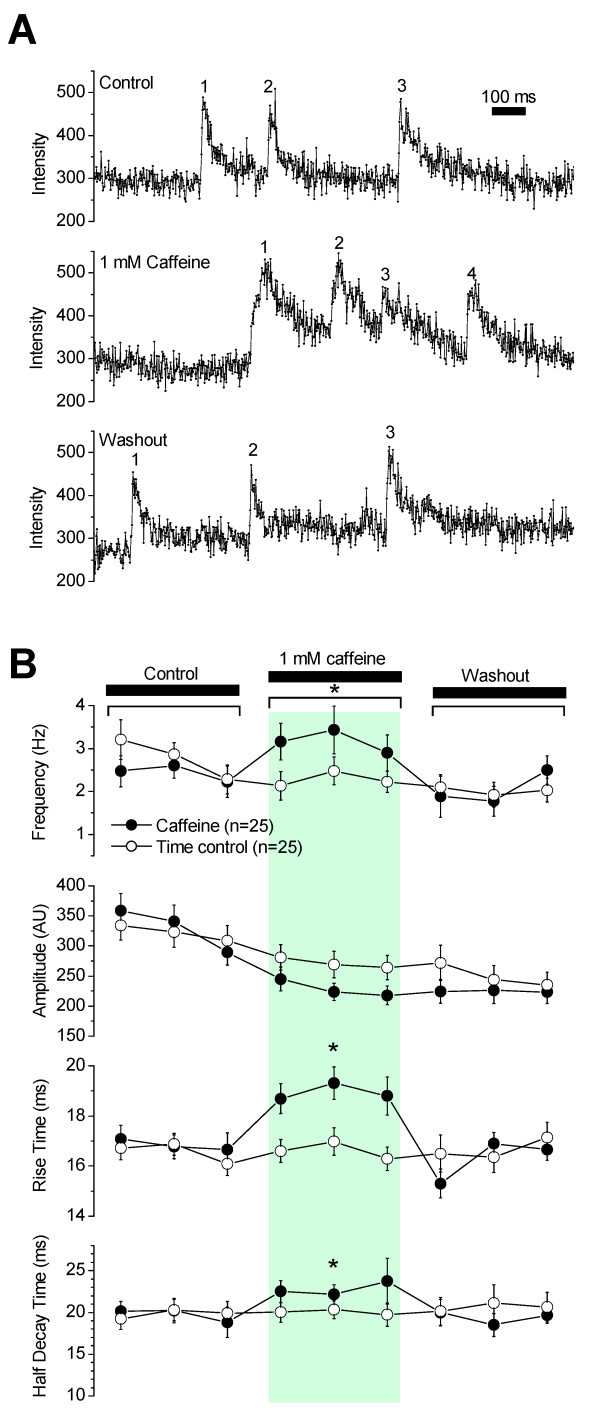
**Caffeine altered frequency, rise and decay times of Ca^2+ ^sparks**. **(A) **Original recordings of Ca^2+ ^sparks in the absence (top trace), presence (middle trace) and washout (bottom trace) of 1 mM caffeine. Numbers beside transients indicate sparks. **(B) **Caffeine increased the frequency, rise and decay time of Ca^2+ ^sparks but had no effect on Ca^2+ ^spark amplitude. Recordings in the presence of caffeine were made at 10, 40 and 70s after drug application and recordings during washout were made at 10, 40 and 70s after termination of drug application. Time control experiments revealed that Ca^2+ ^spark frequency, amplitude, rise time and time to half-decay were reasonably stable for nine 5 s line scans spread over 225 s.

Caffeine is an activator of RyRs, which at very low concentrations (μM - 1 mM) causes an increase in spark frequency whereas when used in high concentrations (> 5 mM) induces a robust Ca^2+ ^release that depletes the Ca^2+ ^stores [4]. Consistent with previous reports of the effects of low concentrations of caffeine, 1 mM caffeine induced significant reversible increases in Ca^2+ ^spark frequency, rise time and time to half-decay in the smooth muscle cells of the SMA (Figure 4). Spark frequency was 3.3 ± 0.4 Hz/spark site in caffeine vs. 2.3 ± 0.2 Hz/spark site in time-matched controls without caffeine (n = 25). Rise time was 19 ± 0.5 ms (1186 sparks from n = 25 sites) in caffeine vs. 17 ± 0.3 ms (1618 sparks from n = 25 sites) in time-matched controls. Time to half-decay was 23 ± 1 ms (1186 sparks from n = 25 sites) in caffeine vs. 20 ± 1 ms (1618 sparks from n = 25 sites) in time-matched controls. In addition to measured spark attributes, spark morphology was altered significantly (Figure 4A). In the presence of caffeine, many sparks exhibited a slower, more rounded rise that lingered at the peak longer than normal sparks, suggesting altered kinetics of RyRs due to activation by caffeine.

### Effect of membrane potential depolarization on Ca^2+ ^sparks

**Figure 5:**
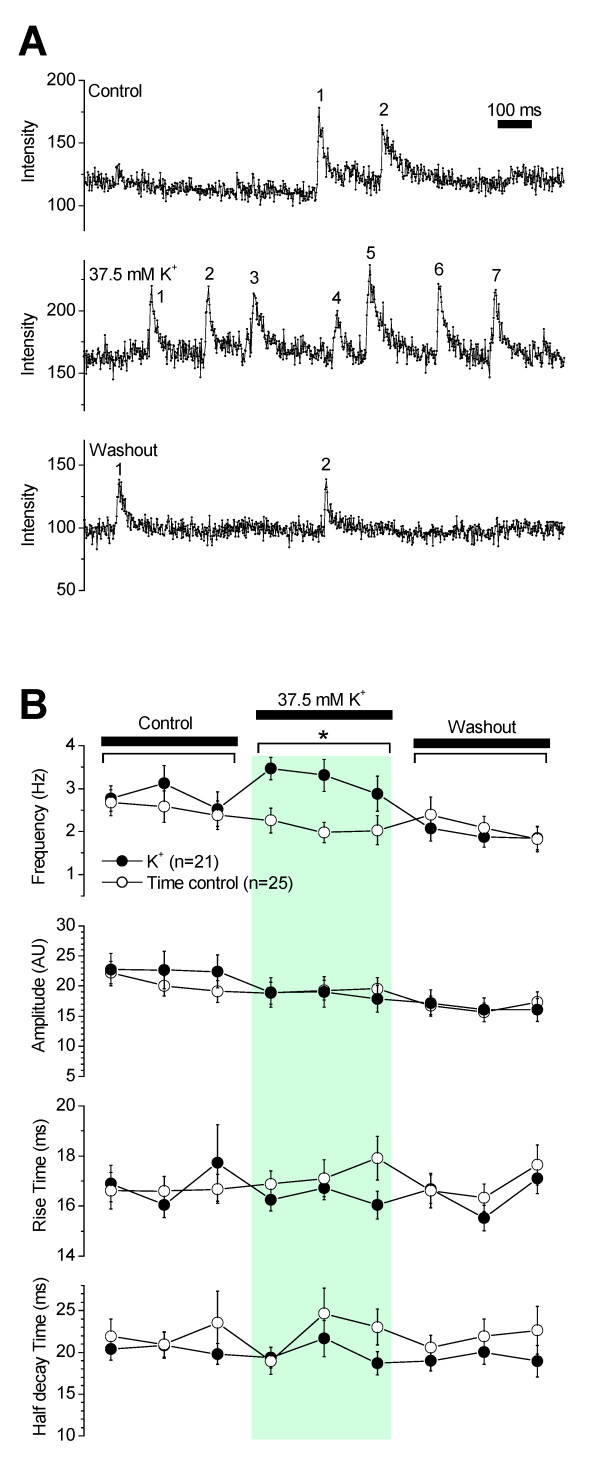
**K^+ ^induced depolarization increased the frequency of Ca^2+ ^sparks**. **(A) **Original recordings of Ca^2+ ^sparks in the absence (top trace), presence (middle trace) and washout (bottom trace) of 37.5 mM K^+^. Numbers beside transients indicate sparks. **(B) **Elevation of extracellular K^+ ^from 3.6 to 37.5 mM increased the frequency of Ca^2+ ^sparks but had no effect on amplitude, rise and half decay times of Ca^2+ ^sparks. Recordings in the presence of 37.5 mM K^+ ^were made at 10, 40 and 70s after K^+ ^application and recordings during washout were made at 10, 40 and 70s after termination of K^+ ^application. Time control experiments are replotted from Figure 3.

Membrane potential depolarization increases intracellular Ca^2+ ^which in turn increases Ca^2+ ^spark frequency in vascular smooth muscle cells [11-13]. Intact SMA segments were depolarized with a 37.5 mM [K^+^] superfusing solution. Consistent with observations in other vascular smooth muscle cells, high K^+ ^significantly increased Ca^2+ ^spark frequency in smooth muscle cells of the SMA that returned to control levels upon re-perfusion with PSS (Figure 5A). Spark frequency in high K^+ ^was 3.2 ± 0.3 Hz/spark site (n = 21 experiments), a 1.4 - fold increase in frequency, with no significant change in spark amplitude, rise time or time to half-decay (Figure 5B).

## Discussion and Conclusion

The SMA, a coiled artery inside the cochlea, is a branch of the anterior inferior cerebellar artery, which in turn branches off from the basilar artery located on the surface of the brain stem [1]. The SMA is a small-caliber vessel (diameter ~ 60 μm) and has a single layer of smooth muscle cells that lacks tightly attached connective tissue [14]. This architecture makes the SMA exquisitely suitable for investigating smooth muscle cell calcium regulation in small vessels (< 70 μm) using intact arteries, as opposed to isolated cells, which are devoid of their natural milieu. The major findings of this study using an intact vessel preparation of the SMA are as follows: (1) The SMA contains ryanodine receptors (RyRs) in the smooth muscle cells. (2) Smooth muscle cells of the SMA exhibit Ca^2+ ^sparks, which are inhibited by ryanodine, an inhibitor of RyR. (3) Elevation of K^+ ^and caffeine increased the frequency of Ca^2+ ^sparks. The kinetics (rise time, decay time and amplitude) were not affected by K^+ ^but were altered by caffeine. These results suggest that RyR-mediated Ca^2+ ^sparks regulate smooth muscle contractility and play a role in the regulation of cochlear blood flow.

Ca^2+ ^sparks, along with BK channel and Ca^2+ ^channel currents play a prominent role in regulating myogenic tone of extra-cerebral arteries on the surface of the brain [5,8]. Ca^2+ ^sparks have been identified in many smooth muscle cells including smooth muscle cells from arteries [8,15,16], portal vein [17], urinary bladder [11,18], airways [19,20] and retinal arterioles [21]. Interestingly, ryanodine-dependent Ca^2+ ^sparks were detected in smooth muscle cells of cremaster feed arteries but not in cremaster arterioles [22]. In this context, the observation of robust ryanodine-sensitive Ca^2+ ^sparks in the smooth muscle cells of the intact SMA, a small-caliber third order branch of the basilar artery, is an important observation (Figure 2).

The gerbil SMA was found to generate well-defined sparks at robust frequencies. Ca^2+ ^sparks occurred at individual spark sites with an average frequency of 2.6 ± 0.1 Hz (Figure 2). This frequency is higher than what has been reported for isolated smooth muscle cells [8,11,15,17,18,20,23] as well as intact cerebral arteries [13] and pressurized mesenteric arteries [16]. Rise time was ~ 17 ms, which is roughly similar to the rise time of 20 ms reported for rat cerebral vessels [8,24]. Time to half decay was ~ 21 ms, which is slightly faster than the 27 ms reported for rat portal vein [17,19] and much faster than 48 - 65 ms reported in cerebral vessels [8,24,25]. In fact, time to half decay of Ca^2+ ^sparks in the gerbil SMA was more similar to the 23 - 37 ms reported for rat heart [26,27]. Reasons for these differences may be due to species differences, due to individuality among blood vessels and differences in experimental conditions.

Ca^2+ ^sparks are regulated by intracellular Ca^2+ ^[4]. In smooth muscle cells, intracellular Ca^2+ ^is strictly regulated by membrane potential. Membrane depolarization by electrical or chemical means or graded increases in intraluminal pressure increases intracellular Ca^2+ ^via Ca^2+ ^influx and Ca^2+ ^release [13,28,29]. Membrane depolarization induced by elevation of K^+ ^has been previously shown to induce vasoconstriction of the SMA, which is sensitive to block of L-type VDCCs [30]. The observation that elevation of K^+ ^increased Ca^2+ ^spark frequency (Figure 5) suggests that Ca^2+ ^sparks play a role in the regulation of smooth muscle excitability and contractility.

In summary, the data presented here establish the intact spiral modiolar artery as an excellent model for the study of RyR-mediated Ca^2+ ^signaling in smooth muscle cells of small arteries (< 70 μm) since it generates high amplitude Ca^2+ ^sparks at robust frequencies. The data suggest a role for Ca^2+ ^sparks in regulating vascular tone of the SMA and cochlear blood flow.

## Methods

### Animal use

Female gerbils (2 - 3 months old) were anesthetized with sodium pentobarbital (100 mg/kg i.p.) and sacrificed by decapitation. All procedures concerning animals were performed under a protocol approved by the Institutional Animal Care and Use Committee at Kansas State University. Temporal bones housing the cochleae were removed, opened, and placed in a chilled (at 4°C) physiological salt solution. The spiral modiolar artery (SMA) is a long vessel (about 7-9 mm in the gerbil) that surrounds and remains loosely coiled against the eighth cranial nerve in the modiolus of the cochlea. The SMA was gently separated from the surrounding nerve and uncoiled taking care not to stretch the artery. Vessels segments were obtained from the center portion of the vessel, avoiding edges that may have been damaged during isolation.

### Bodipy staining

Isolated vessel segments were incubated for 1 hr at RT in PSS containing 0.2% Triton-X and 1 μM Bodipy^®^-ryanodine (Molecular Probes, Eugene OR) with or without 250 μM unlabelled ryanodine. Vessels segments were washed, mounted on slides and observed by confocal microscopy (LSM-510Meta, Carl Zeiss, Germany).

### Vascular superfusion

Isolated vessel segments were mounted on a custom-built superfusion chamber and held with two blunt glass needles operated by joy-stick micromanipulators (MN-151, Narashige, Japan). The bath chamber was installed on the stage of an inverted microscope. Superfusion was set to a rate of 2.5 bath chamber volumes per second. Water jacketing of perfusion lines ensured a constant temperature of 37°C and minimized the formation of air bubbles, which pose a threat to the vessel segment. Vessels were superfused either with a physiological saline solution (PSS) containing (in mM) 150 NaCl, 3.6 KCl, 1 MgCl_2_, 1 CaCl_2_, 5 HEPES, and 5 glucose, pH 7.4 or with a 37.5 mM K^+ ^solution containing 115 NaCl, 37.5 KCl, 1 MgCl_2_, 1 CaCl_2_, 5 HEPES and 5 glucose, pH 7.4. Caffeine was directly dissolved in PSS and ryanodine was solubilized in DMSO before introduction into PSS. The final DMSO concentration was 0.1%. Drugs were purchased from Sigma-Aldrich (St. Louis, MO).

### Measurement of Ca^2+ ^Sparks

Isolated vessel segments were loaded with the Ca^2+ ^indicator dye fluo-4 AM (5 μM, Molecular Probes, Eugene, OR) for 40 minutes at 37°C and mounted in a superfusion chamber fixed on the motorized stage of an inverted microscope (Axiovert 200, Carl Zeiss, Göttingen, Germany). The Ca^2+ ^indicator dye fluo-4 is an analog of the commonly used fluo-3 that provides higher signal levels in response to excitation with the 488 nm line of the argon laser (Molecular Probes Product Information Sheet). Superfusion was carried out as described above. Fluo-4 was excited by the argon laser (488 nm) operated at 9 mW (30% of the maximum power of 30 mW) that was attenuated by an acousto-optical tunable filter to 1% for the measurement of Ca^2+ ^sparks (LSM-Meta, Carl Zeiss, Göttingen, Germany). The beam path consisted of a notch filter (488 nm) followed by two long-pass filters (490 and 505 nm) and an oil immersion objective (Plan-Neofluar 40x, 1.3 NA, Carl Zeiss). Ca^2+ ^sparks were imaged in confocal xy scans (76.8 × 5.2 μm corresponding to 512 × 35 pixels) with a spatial (x,y,z) resolution of 0.15 × 0.15 μm spatial resolution, an optical depth <0.9 μm and a temporal resolution of 41.5 ms (Figure 2A). For detailed analysis of spark kinetics, Ca^2+ ^sparks were recorded in 5s non-confocal (open pinhole) line scans. Line scans, defined as repeated sweeps of the scanner along a single line (76.8 μm corresponding to 512 pixels), were performed at a chosen spark site with a spatial (x,z) resolution of 0.15 × <12.4 μm and a temporal resolution of 1.92 ms. Nine consecutive line scans, each lasting 5s, were taken. The time interval between line scans was ~40 s between the first and the second scan and ~25 s between all further scans.

### Ca^2+ ^spark detection and analysis

Line scan images (512 × 2605 pixels), covering 76.8 μm for 5 s, were visually inspected for sparks. Intensity values covering the width of a spark site were averaged and exported to a text file ('raw data') for analysis using an algorithm written in Labtalk (Origin 6.0, OriginLab). Frequency, amplitude, rise time (0 - 100%) and time to half-decay were determined for individual spark sites (Figure 2).

The analysis algorithm used the 'raw' data as well as a 'subtracted' data set that was generated by subtraction of a 'baseline' dataset generated by a 601-point sliding window average from a 'smoothed' dataset generated by a 5-point sliding window average of the 'raw' data. The 'subtracted' data were used for the identification of Ca^2+ ^sparks. Sparks were identified by a user-defined number of consecutive intensity increases that spanned a user-defined minimum amplitude. 'Subtracted' data were further used to evaluate whether a spark is suitable for determining amplitude, rise and decay times. Suitable sparks are sparks that arise near or below the baseline (Figure 2C). Sparks that follow a previous spark too closely arise from a higher value which could lead to an underestimate of their amplitude (Figure 2D). Amplitude was determined as difference between the raw data peak and the base of the spark. The base was determined as the average of 10 raw data points immediately prior to the first of the consecutive increases. The rise time was the time from the end of the base to the peak and the time to half decay was the time between the peak and the time when the 'averaged' data decayed by half of the amplitude. The 'raw' data were graphed and annotated for visual inspection (Figure 2). Each identified spark was marked by a number and by a vertical indicator line transecting the peak of the spark. Measurable sparks received additional annotation including a horizontal line segment marking the base of the spark and a vertical line transecting the time of half decay. Measurements of amplitude, rise time and time to half-decay were averages of data that passed visual inspection.

### Statistics

Frequency, amplitude, rise and half-decay times of Ca^2+ ^sparks were graphed as averages of values during each of the nine sequential measurements. Ca^2+ ^spark data reported in the text are averages of three sequential measurements. Error bars are SEM. Significance was assumed at p < 0.05 and determined by either paired or unpaired t-test, as appropriate.

## Authors' contributions

GK, KR, EQS and PW wrote the manuscript. KR collected and analyzed Ca^2+ ^sparks data, developed the software code for sparks analysis. SB performed the Bodipy-ryanodine staining. PW conceived of the study and participated in the design of experiments and development of the analysis software. All authors have read and agreed to the final version of the manuscript.

## References

[B1] Axelsson A Comparative anatomy of cochlear blood vessels Am J Otolaryngol 1988 9 278 290 10.1016/S0196-0709(88)80036-X 3067591

[B2] Wangemann P Liu J Marcus DC Ion transport mechanisms responsible for K+ secretion and the transepithelial voltage across marginal cells of stria vascularis in vitro Hear Res 1995 84 19 29 10.1016/0378-5955(95)00009-S 7642451

[B3] Davis MJ Hill MA Signaling mechanisms underlying the vascular myogenic response Physiol Rev 1999 79 387 423 10221985 10.1152/physrev.1999.79.2.387

[B4] Cheng H Lederer WJ Calcium sparks Physiol Rev 2008 88 1491 1545 10.1152/physrev.00030.2007 18923188

[B5] Knot HJ Standen NB Nelson MT Ryanodine receptors regulate arterial diameter and wall [Ca2+] in cerebral arteries of rat via Ca2+-dependent K+ channels J Physiol 1998 508 Pt 1 211 221 9490841 10.1111/j.1469-7793.1998.211br.x PMC2230867

[B6] Bolton TB Imaizumi Y Spontaneous transient outward currents in smooth muscle cells Cell Calcium 1996 20 141 152 10.1016/S0143-4160(96)90103-7 8889205

[B7] Jaggar JH Wellman GC Heppner TJ Porter VA Perez GJ Gollasch M Kleppisch T Rubart M Stevenson AS Lederer WJ Ca2+ channels, ryanodine receptors and Ca(2+)-activated K+ channels: a functional unit for regulating arterial tone Acta Physiol Scand 1998 164 577 587 10.1046/j.1365-201X.1998.00462.x 9887980

[B8] Nelson MT Cheng H Rubart M Santana LF Bonev AD Knot HJ Lederer WJ Relaxation of arterial smooth muscle by calcium sparks Science 1995 270 633 637 10.1126/science.270.5236.633 7570021

[B9] Wonneberger K Scofield MA Wangemann P Evidence for a calcium-sensing receptor in the vascular smooth muscle cells of the spiral modiolar artery J Membr Biol 2000 175 203 212 10.1007/s002320001068 10833530

[B10] Li L Ma KT Zhao L Si JQ Niflumic acid hyperpolarizes the smooth muscle cells by opening BK(Ca) channels through ryanodine-sensitive Ca(2+) release in spiral modiolar artery Sheng Li Xue Bao 2008 60 743 750 19082430

[B11] Collier ML Ji G Wang Y Kotlikoff MI Calcium-induced calcium release in smooth muscle: loose coupling between the action potential and calcium release J Gen Physiol 2000 115 653 662 10.1085/jgp.115.5.653 10779321 PMC2217224

[B12] Jaggar JH Porter VA Lederer WJ Nelson MT Calcium sparks in smooth muscle Am J Physiol Cell Physiol 2000 278 C235 C256 10666018 10.1152/ajpcell.2000.278.2.C235

[B13] Jaggar JH Stevenson AS Nelson MT Voltage dependence of Ca2+ sparks in intact cerebral arteries Am J Physiol 1998 274 C1755 C1761 9611142 10.1152/ajpcell.1998.274.6.C1755

[B14] Wangemann P Cohn ES Gruber DD Gratton MA Ca2+-dependence and nifedipine-sensitivity of vascular tone and contractility in the isolated superfused spiral modiolar artery in vitro Hear Res 1998 118 90 100 10.1016/S0378-5955(98)00017-3 9606064

[B15] Furstenau M Lohn M Ried C Luft FC Haller H Gollasch M Calcium sparks in human coronary artery smooth muscle cells resolved by confocal imaging J Hypertens 2000 18 1215 1222 10.1097/00004872-200018090-00007 10994752

[B16] Miriel VA Mauban JR Blaustein MP Wier WG Local and cellular Ca2+ transients in smooth muscle of pressurized rat resistance arteries during myogenic and agonist stimulation J Physiol 1999 518 Pt 3 815 824 10420017 10.1111/j.1469-7793.1999.0815p.x PMC2269448

[B17] Gordienko DV Bolton TB Crosstalk between ryanodine receptors and IP(3) receptors as a factor shaping spontaneous Ca(2+)-release events in rabbit portal vein myocytes J Physiol 2002 542 743 762 10.1113/jphysiol.2001.015966 12154176 PMC2290443

[B18] Herrera GM Heppner TJ Nelson MT Voltage dependence of the coupling of Ca(2+) sparks to BK(Ca) channels in urinary bladder smooth muscle Am J Physiol Cell Physiol 2001 280 C481 C490 11171567 10.1152/ajpcell.2001.280.3.C481

[B19] Mironneau J Arnaudeau S Macrez-Lepretre N Boittin FX Ca2+ sparks and Ca2+ waves activate different Ca(2+)-dependent ion channels in single myocytes from rat portal vein Cell Calcium 1996 20 153 160 10.1016/S0143-4160(96)90104-9 8889206

[B20] Liu QH Zheng YM Wang YX Two distinct signaling pathways for regulation of spontaneous local Ca2+ release by phospholipase C in airway smooth muscle cells Pflugers Arch 2007 453 531 541 17093969 10.1007/s00424-006-0130-1

[B21] Curtis TM Tumelty J Dawicki J Scholfield CN McGeown JG Identification and spatiotemporal characterization of spontaneous Ca2+ sparks and global Ca2+ oscillations in retinal arteriolar smooth muscle cells Invest Ophthalmol Vis Sci 2004 45 4409 4414 10.1167/iovs.04-0719 15557449 PMC2590679

[B22] Westcott EB Jackson WF Heterogeneous function of ryanodine receptors, but not IP3 receptors, in hamster cremaster muscle feed arteries and arterioles Am J Physiol Heart Circ Physiol 2011 300 H1616 H1630 10.1152/ajpheart.00728.2010 21357503 PMC3094085

[B23] Gordienko DV Bolton TB Cannell MB Variability in spontaneous subcellular calcium release in guinea-pig ileum smooth muscle cells J Physiol 1998 507 Pt 3 707 720 9508832 10.1111/j.1469-7793.1998.707bs.x PMC2230821

[B24] Perez GJ Bonev AD Patlak JB Nelson MT Functional coupling of ryanodine receptors to KCa channels in smooth muscle cells from rat cerebral arteries J Gen Physiol 1999 113 229 238 10.1085/jgp.113.2.229 9925821 PMC2223357

[B25] Bonev AD Jaggar JH Rubart M Nelson MT Activators of protein kinase C decrease Ca2+ spark frequency in smooth muscle cells from cerebral arteries Am J Physiol 1997 273 C2090 C2095 9435516 10.1152/ajpcell.1997.273.6.C2090

[B26] Cheng H Lederer WJ Cannell MB Calcium sparks: elementary events underlying excitation-contraction coupling in heart muscle Science 1993 262 740 744 10.1126/science.8235594 8235594

[B27] Lukyanenko V Gyorke I Gyorke S Regulation of calcium release by calcium inside the sarcoplasmic reticulum in ventricular myocytes Pflugers Arch 1996 432 1047 1054 10.1007/s004240050233 8781199

[B28] Knot HJ Nelson MT Regulation of arterial diameter and wall [Ca2+] in cerebral arteries of rat by membrane potential and intravascular pressure J Physiol 1998 508 Pt 1 199 209 9490839 10.1111/j.1469-7793.1998.199br.x PMC2230857

[B29] Kamishima T McCarron JG Regulation of the cytosolic Ca2+ concentration by Ca2+ stores in single smooth muscle cells from rat cerebral arteries J Physiol 1997 501 Pt 3 497 508 9218210 10.1111/j.1469-7793.1997.497bm.x PMC1159451

[B30] Wangemann P Gruber DD The isolated in vitro perfused spiral modiolar artery: pressure dependence of vasoconstriction Hear Res 1998 115 113 118 10.1016/S0378-5955(97)00184-6 9472740

